# How does human-AI collaboration task complexity affect employee work engagement? The roles of humble leadership and AI self-efficacy

**DOI:** 10.3389/fpsyg.2026.1767967

**Published:** 2026-02-10

**Authors:** Bolin Wang, Simeng Liu, Chenhao Luo

**Affiliations:** Changsha Normal University, College of Economics and Management, Changsha, China

**Keywords:** AI self-efficacy, HAI-C task complexity, HAI-C tech-learning anxiety, humble leadership, work engagement

## Abstract

**Introduction:**

With the rapid advancement of artificial intelligence (AI) technology, human-AI collaboration has become increasingly prevalent in workplaces, profoundly impacting employees’ psychology and behavior. Based on the Job Demands-Resources (JD-R) theory, this study examines the effects of human-AI collaboration task complexity (HAI-C task complexity) on employees’ work engagement, with human-AI collaboration tech-learning anxiety (HAI-C tech-learning anxiety) as a mediator, and explores the moderating roles of humble leadership and AI self-efficacy.

**Methods:**

This study employed a three-wave longitudinal survey design to collect matched data from 497 employees. Hierarchical regression analysis, along with bootstrapping methods, was employed for empirical testing.

**Results:**

The findings indicate that HAI-C task complexity negatively affects employees’ work engagement by amplifying their HAI-C tech-learning anxiety. AI self-efficacy can mitigate this negative indirect impact of HAI-C task complexity on work engagement. Humble leadership indirectly alleviates this negative indirect effect by enhancing employees’ AI self-efficacy.

**Discussion:**

The findings reveal the inhibitory effect of HAI-C task complexity on employees’ work engagement. From the two dimensions of job resources and personal resources, it explores corresponding mitigation mechanisms, as well as the contextual and psychological intervention mechanisms involved in how individuals evaluate job demands. This provides novel theoretical perspectives and practical implications for understanding the practical value of human-AI collaboration in organizational contexts and for enhancing employees’ work engagement within human-AI collaboration frameworks.

## 1 Introduction

In recent years, with the widespread application of artificial intelligence (AI) technology in corporate workplaces (Yu X. et al., 2023), AI has gradually transitioned from being merely a “tool” to becoming a “collaborative partner,” giving rise to the work mode known as “human-AI collaboration” (Kong et al., 2023). Human-AI collaboration refers to the process where employees and AI interact and cooperate to jointly complete work tasks (Sowa et al., 2021; Xu et al., 2025). In this mode, employees and AI focus on their respective strengths to engage in “complementary collaboration”: for instance, AI specializes in handling complex computations and analyzing massive datasets, while humans concentrate on complex decision-making, applying social and emotional skills, and executing precise operations (Sowa et al., 2021). This model not only significantly reduces human operational errors but also enhances work efficiency, decision-making capabilities, and innovation capacity (Malik et al., 2021; Makarius et al., 2020; Qin et al., 2023; Pereira et al., 2023).

Research shows that as AI becomes increasingly embedded in organizational operations, the human-AI collaboration model empowers employees while profoundly altering traditional work content and business processes (Umair et al., 2023; Li et al., 2022). This inevitably increases the complexity of work content, primarily manifested in the inherent difficulty of tasks related to human-AI collaboration, the cognitive load employees must bear, and the problem-solving abilities required to complete their work (Wu et al., 2024; Cai et al., 2025; Dong et al., 2025). Consequently, employees may face higher learning demands and increased pressure to update their skills (Choudhary et al., 2023; Sharma et al., 2023). A study by Oracle Corporation revealed that after introducing AI technology, 51% of the company’s employees exhibited anxiety due to their inability to adapt to and mastering these technologies (Blanchard, 2018). Therefore, in the context of human-AI collaboration, whether the increased complexity of work content leads to anxiety among employees, whether such emotions reduce work engagement, and, more critically, how to effectively mitigate this impact have become urgent and significant issues to address at this stage.

In recent years, research on human-AI collaboration has gradually increased. Some scholars argue that human-AI collaboration can significantly optimize work processes and enhance job performance, productivity, work efficiency, proactive behavior, learning behavior, and creativity (Wang M. et al., 2023; Liu and Li, 2025; Paluch et al., 2022; Przegalinska et al., 2025; Wu and Zhang, 2024; Sun et al., 2025; Zhang et al., 2025; Jia et al., 2024; Yin et al., 2024). In contrast, other scholars contend that human-AI collaboration may exacerbate employees’ counterproductive work behavior, work alienation, job insecurity, knowledge hiding behavior, and unethical conduct (Meng et al., 2025; Bai and Zhang, 2025; Kim and Kim, 2024; Liang et al., 2025; Kim et al., 2022). Although existing studies provide a foundation for understanding the impact of human-AI collaboration on organizations and employees, there remains room for further expansion in this field. First, as human-AI collaboration increasingly becomes the norm in workplaces, there is a relative lack of exploration into whether the resulting task complexity triggers employee anxiety and affects work engagement. This perspective is crucial for a deeper understanding of the practical value of human-AI collaboration within organizations (Wu et al., 2024; Cai et al., 2025). Second, research on the negative impacts of human-AI collaboration often focuses on the mechanisms underlying such behaviors (He et al., 2025; Hai et al., 2025), while frequently neglecting organizational and individual-level coping strategies and interventions. This theoretical imbalance also limits the comprehensiveness of human-AI collaboration management practices. Third, leadership, as a key factor influencing employee psychology and behavior, has not yet been sufficiently explored in its role within the relationship between human-AI collaboration and individual behavior, warranting further investigation (Bakker and Demerouti, 2017). In recent years, as AI technology has continuously reshaped work environments, traditional leadership models are facing fundamental challenges. The introduction of AI not only influences employees’ psychology and behavior but also imposes new requirements on leaders’ role positioning and behavioral patterns, prompting them to reevaluate their roles and adjust management strategies (Fountaine et al., 2019; Peifer et al., 2022; Fousiani et al., 2024). Multiple studies have pointed out that in addressing the changes and challenges brought about by AI, humility is increasingly emerging as a critical trait for leaders in the new technological landscape (Guo et al., 2025; Rego et al., 2019). Humble leadership is a style oriented toward others and emphasizes bottom-up interactions (Owens and Hekman, 2012; Kelemen et al., 2023). Compared to traditional top-down management models, this leadership style often demonstrates greater adaptability in highly dynamic and uncertain environments (Rego et al., 2019) and has also been proven more effective in alleviating employee anxiety and reducing psychological stress (Edmondson and Lei, 2014; Owens and Hekman, 2016). Therefore, against the backdrop of continuous AI integration and rapid technological iteration in modern enterprises, exploring the interactive effects of humble leadership and human-AI collaboration task complexity (HAI-C task complexity) on employees holds significant academic value and practical relevance.

According to the Job Demands-Resources (JD-R) theory, job characteristics can be divided into two main categories: job demands and job resources. Job demands refer to aspects of work that require sustained physical or mental effort from employees, leading to the depletion of their physiological and psychological resources. This may trigger negative emotions, such as anxiety, thereby undermining their intrinsic motivation related to work tasks. Conversely, job resources are organizational or personal factors that help employees achieve work goals and reduce the physiological and psychological costs associated with job demands. These resources can effectively buffer the impact of job demands on individual anxiety (Demerouti et al., 2001; Ong and Johnson, 2023; Bakker and Demerouti, 2017). This study posits that, in the human-AI collaboration context, individuals experience two psychological processes. On one hand, when employees face complex tasks related to human-AI collaboration, they often experience fear and worry due to the need to learn advanced and complex knowledge and technologies (Wu et al., 2024; Bauer et al., 2023; Tursunbayeva and Renkema, 2023). Concerns about whether they can master the skills required for effective human-AI collaboration further diminish their sense of work engagement. On the other hand, humble leadership, as a key job resource, is characterized by actively listening to employees’ ideas, valuing others’ contributions, and being adept at unlocking subordinates’ potential (Owens and Hekman, 2012; Owens et al., 2013). These unique behavioral traits strengthen employees’ psychological safety and reduce negative emotions (Qian et al., 2020; Wang and Zhou, 2021; Basford et al., 2014), thereby mitigating the strain induced by job demands (Niu et al., 2025). Simultaneously, self-efficacy, as a typical personal resource, has been proven to buffer the impact of job demands on individual strain (Schaufeli and Taris, 2014; Hong, 2022). Recent research indicates that, compared to generalized self-efficacy, specific self-efficacy within a particular context (such as AI self-efficacy) often yields more robust predictive effects and is better suited to meet the resource-buffering needs of specific task scenarios (Rayburn et al., 2021).

Based on the above analysis, this study applies the JD-R theoretical framework, with human-AI collaboration tech-learning anxiety (HAI-C tech-learning anxiety) as a mediating variable, humble leadership and AI self-efficacy as moderating variables, to construct a theoretical model of how HAI-C task complexity influences work engagement. This aims to deepen theoretical research and academic dialog on this topic. Furthermore, this study makes several theoretical contributions to existing research: first, it focuses on a relatively under-explored mechanism of how HAI-C task complexity affects employee psychology and behavior, thereby deepening the theoretical understanding of human-AI collaboration. Second, grounded in the JD-R model, this study explores the intervention and defensive mechanisms against the negative effects of human-AI collaboration through the dimensions of job resources and personal resources (Bakker and Demerouti, 2017). This enriches the application of the JD-R model within the human-AI collaboration field and offers practical implications for management. Third, the findings of this study reveal that HAI-C task complexity can be simultaneously motivating and draining in different contexts, this conclusion deepens our understanding of the psychological intervention mechanisms involved in how individuals evaluate job demands, thereby further advancing the integration and development of the JD-R theory and the challenge-hindrance stressor framework. Fourth, by introducing humble leadership—an important leadership style—into the domain of human-AI collaboration and defining it as an effective job resource, this study enriches research on the relationship between leadership, human-AI collaboration, and employee behavior. Finally, this study introduces “AI self-efficacy” as a core variable, confirming not only its moderating and buffering roles in the mechanism by which HAI-C task complexity affects employee work engagement, but also revealing its interrelationship with humble leadership, as well as the internal mechanisms through which both jointly alleviate strain, which provides an important addition to the discussion in the JD-R theory on how job resources activate personal resources.

## 2 Theory and hypotheses

To construct the analytical framework for this study, we adopted a semi-systematic review approach. The rationale for employing this method in the literature review is based on several key considerations. First, this approach is well-suited for theoretical integration across interdisciplinary and rapidly evolving fields. Given that the topic of this study spans multiple domains, including AI, management, organizational behavior, and applied psychology, utilizing this method allows for more effective identification and integration of key concepts, theoretical perspectives, and empirical findings from these diverse fields. Second, the purpose of this study is not to conduct a comprehensive meta-analysis on a narrowly defined issue, which would be more appropriate for a systematic review, but rather to synthesize existing literature, derive theoretical insights, and construct a theoretical model (Snyder, 2019).

### 2.1 Job demand path: the mediating role of HAI-C tech-learning anxiety

#### 2.1.1 HAI-C task complexity and HAI-C tech-learning anxiety

HAI-C task complexity refers to the perceived challenges in knowledge and skills that employees encounter when collaborating with AI to carry out tasks. This is specifically reflected in aspects such as task unpredictability, significantly increased knowledge requirements, and the cognitive demands related to human-AI collaboration (Dong et al., 2025; Verma and Singh, 2022). Research indicates that with the continuous iteration of AI technology, employees who work in collaboration with AI, their original work content and processes may be reshaped, and traditional tasks will face significant impact from technological changes (Nam, 2019; Umair et al., 2023; Zhang and Jin, 2023). This increase in task complexity places higher demands on employees’ knowledge and skills (Choudhary et al., 2023; Bauer et al., 2023).

Compared to previous technologies, such as automation equipment and basic information systems, AI technology exhibits significant differences in technical attributes and application scenarios. Traditional technologies typically follow preset, deterministic rules and processes, displaying a “command-execution” characteristic with fixed functional boundaries and standardized operational logic. Employees only need to master established procedures to complete collaboration, with the corresponding learning requirements largely focused on “operational proficiency” (Aghion et al., 2017). In contrast, AI technology possesses three key characteristics: autonomous decision-making, dynamic adaptability, and decision opacity (Berente et al., 2021). Autonomous decision-making refers to the ability of AI systems to independently analyze, judge, and execute tasks or provide decision recommendations based on preset objectives, real-time data, and internal models without direct human intervention (Raisch and Krakowski, 2021). Dynamic adaptability means that AI systems (especially those based on machine learning) can continuously optimize their models and behavioral performance as new data is input and environmental feedback is received (Berente et al., 2021). Decision opacity refers to the phenomenon in which the internal decision-making processes of an AI model are highly complex, nonlinear, and opaque, making it difficult for people to clearly explain why the system produces a specific output given a particular input (Davenport and Ronanki, 2018). These characteristics mean that when collaborating with AI, employees face not only procedural complexity but also high cognitive demands and adaptive challenges arising from the technological features of AI. This involves the process of effectively collaborating and working with an intelligent system capable of continuous learning, potentially autonomous adjustment, and possessing decision-making logic that is not fully transparent (Kellogg et al., 2020).

HAI-C tech-learning anxiety refers to the fear and apprehension experienced by employees when required to learn advanced and complex artificial intelligence technologies and to collaborate effectively with AI. This anxiety is a common negative psychological state (Wu et al., 2024). According to the JD-R model, increased job demands deplete employees’ physical, emotional, and cognitive resources, impair their self-regulation abilities, and reduce the resources available to cope with future job demands, thereby triggering anxiety and fatigue (Demerouti et al., 2001), and even leading to serious health issues (Bakker and Demerouti, 2024). Within the JD-R framework, task complexity is often regarded as a significant job demand (Demerouti et al., 2001). When employees face complex task requirements arising from human-AI collaboration—such as the adoption of new AI-related technologies or reengineering and optimization of work processes (Verma and Singh, 2022; Nawaz et al., 2024)—they may question their capability to learn the necessary skills due to the cognitive stress brought by AI technological development or confusion about the human-AI collaboration mode, thereby inducing anxiety about learning advanced AI technologies (Jiang et al., 2021). This is particularly true when they perceive themselves as lacking sufficient ability, adequate support, or when facing uncertainty (Yin et al., 2024; Stein et al., 2020). Such anxiety may further lead to employees’ resistance or reluctance to learning skills and technologies related to human-AI collaboration. Based on this, the following hypothesis is proposed:

*Hypothesis* 1: HAI-C task complexity is positively related to HAI-C tech-learning anxiety.

#### 2.1.2 HAI-C tech-learning anxiety and work engagement

Work engagement refers to a positive work state of mind in which individuals are fully integrated into their work roles (Schaufeli et al., 2002). It encompasses three dimensions: vigor, dedication, and absorption. Vigor is characterized by high levels of energy and mental resilience while working, dedication involves a strong identification with and sense of significance in one’s work, and absorption is the state of being fully concentrated and happily engrossed in work activities (Bakker and Demerouti, 2024). As one of the earliest concepts integrated into the JD-R model, work engagement has been established as a significant outcome variable of individual stress and possesses a solid theoretical foundation within the JD-R framework (Schaufeli and Bakker, 2004; Bakker and Demerouti, 2017). However, within research related to human-AI collaboration, its application requires further examination and validation.

According to the JD-R model, individuals must continuously obtain sufficient physical and psychological resources to maintain a high level of work engagement (Bakker and Demerouti, 2008). HAI-C tech-learning anxiety, as a negative psychological state, consumes individual resources (Wu et al., 2024). When employees experience specific anxiety due to learning advanced and complex technological skills, they often develop feelings of helplessness and fear, which weakens their ability and personal resources to cope with stressors (Bakker and Demerouti, 2017). Consequently, it becomes challenging for them to achieve a state of engagement characterized by vigor, dedication, and absorption at work (Bakker and Demerouti, 2008). Simultaneously, this anxious state may lead individuals to adopt a negative attitude toward learning skills or methods related to human-AI collaboration (Wang and Wang, 2019), and hinder their willingness and ability to proactively seek help or consult others about relevant issues (Tannenbaum and Wolfson, 2022). This may further deplete personal resources and trigger a resource loss spiral (Wu et al., 2024), ultimately resulting in a decline in work proactivity (Bakker and Demerouti, 2017; Aguiar-Quintana et al., 2021). Based on this, the following hypothesis is proposed:

*Hypothesis* 2: HAI-C tech-learning anxiety is negatively related to work engagement.

#### 2.1.3 The mediating role of HAI-C tech-learning anxiety

In the workplace, HAI-C task complexity constitutes a job demand characterized by a high cognitive load and high adaptive burden, consistently challenging employees’ existing knowledge structures, skill sets, and learning capabilities (Schiavo et al., 2024; Wang et al., 2024). When such complexity exceeds an individual’s current capacity or adaptive resilience, it tends to trigger HAI-C tech-learning anxiety (Wu et al., 2024). This anxiety includes concerns about failing to master new technologies, fear of performance lagging behind, doubts about one’s ability to handle human-AI collaboration effectively, as well as feelings of losing control and experiencing burnout amid rapid changes (Stein et al., 2020; Yin et al., 2024). From the perspective of the JD-R model, this anxious state constitutes an additional endogenous psychological demand. It requires individuals to mobilize greater emotional and cognitive resources to cope, thereby depleting their psychological energy and intensifying psychological fatigue (Ong and Johnson, 2023). This further diminishes the deep-level resources available for focus, vigor, and dedication, ultimately leading to a decline in work engagement (Bakker and Demerouti, 2024). Based on this, the following hypothesis is proposed:

*Hypothesis* 3: HAI-C tech-learning anxiety plays a negative mediating role between HAI-C task complexity and work engagement.

### 2.2 Job resource buffer process: the moderating role of humble leadership

Humble leadership, as a formal academic concept, was first proposed by Owens and Hekman (2012). Through ongoing scholarly exploration of its connotation and structure (Zheng and Ahmed, 2024), a consensus definition has emerged: humble leadership is essentially a bottom-up leadership model, with its core connotation comprising three dimensions (Chandler et al., 2023; Zhang et al., 2024). First, accurate self-awareness, which refers to leaders being able to objectively examine their own limitations and shortcomings, thereby showing more empathy rather than solely blaming subordinates when facing deficiencies. Second, appreciation of others, meaning valuing the contributions and worth of others, and being adept at uncovering the potential of subordinates. Third, openness to learning, demonstrated through a receptive attitude toward new ideas, perspectives, and suggestions (Owens et al., 2013; Shen et al., 2025). Research indicates that humble leadership can enhance employees’ affective commitment, affective trust, job satisfaction, psychological safety, and self-efficacy, among other factors (Wang et al., 2022; Owens et al., 2013; Wang and Zhou, 2021; Qian et al., 2020).

According to the JD-R theory, job resources can mitigate the impact of job demands on employee strain (Demerouti et al., 2001; Bakker and Demerouti, 2017). Job resources refer to organizational or psychological factors in the workplace that help employees achieve work goals, reduce the physical and psychological costs associated with job demands, and promote personal growth, learning, and development (Bakker, 2011; Bakker and Demerouti, 2007). Existing research has confirmed that leadership is a significant provider of individual resources within organizations (Bakker and Demerouti, 2017).

As a bottom-up leadership model, humble leadership provides multidimensional key job resources that effectively buffer the pathways through which job demands trigger anxiety (Bakker and Demerouti, 2017). Its core traits—such as openness, inclusivity, empathy, appreciation for others’ contributions, emphasis on learning and growth, and a focus on unlocking employee potential—can translate into a scarce contextual resource (Chandler et al., 2023; Zhang et al., 2024). This resource is directly infused into employees’ processes for coping with complex AI tasks. Specifically, humble leadership first fosters a psychologically safe environment (Qian et al., 2020; Wang and Zhou, 2021), treating employees’ confusion and trial-and-error in human-AI collaboration as a normal part of the learning process. This inclusivity significantly reduces employees’ fear and anxiety about exposing their inadequacies, allowing highly complex task challenges to be reassessed as developmental opportunities rather than personal threats. Second, through equal and sincere communication and collaborative problem-solving (Nguyen et al., 2020), humble leadership helps employees deconstruct the uncertainties associated with HAI-C complex tasks, providing targeted guidance or seeking external support, thereby alleviating employees’ cognitive overload and sense of helplessness. Third, the empathy demonstrated by humble leadership enables employees to feel emotional support and a sense of belonging when facing technological pressure (Basford et al., 2014). This emotional resource directly counteracts the tension and anxiety triggered by task demands. Finally, by advocating a growth mindset and promoting team collaboration (Liborius and Kiewitz, 2022; Ma et al., 2019), humble leadership transforms the sense of isolation individuals may feel when dealing with complex AI systems into collective learning and problem-solving efficacy, further dispersing sources of stress. Therefore, in contexts with strong humble leadership, employees possess sufficient resource reserves to understand and cope with tasks, even when facing highly complex AI collaboration challenges. This significantly weakens the positive impact of task complexity on learning anxiety. Conversely, in contexts lacking humble leadership, resource scarcity means any increase in task complexity more directly translates into heightened employee anxiety. Based on this, the following hypothesis is proposed:

*Hypothesis* 4a: Humble leadership negatively moderates the relationship between HAI-C task complexity and HAI-C tech-learning anxiety negatively.

*Hypothesis* 4b: Humble leadership negatively moderates the mediation of HAI-C tech-learning anxiety between HAI-C task complexity and work engagement.

### 2.3 Personal resources buffer process: the moderating role of AI self-efficacy

According to the JD-R theory, personal resources are defined as positive self-evaluations, referring to individuals’ perceptions of their own ability to successfully control and influence their environment (Hobfoll et al., 2003). Within the JD-R framework, personal resources function similarly to job resources (Bakker and Demerouti, 2017). Employees’ self-efficacy, as a personal resource, has been demonstrated to buffer the impact of job demands on burnout and to enhance the positive effects of other job resources on work engagement (Schaufeli and Taris, 2014).

**Figure 1:**
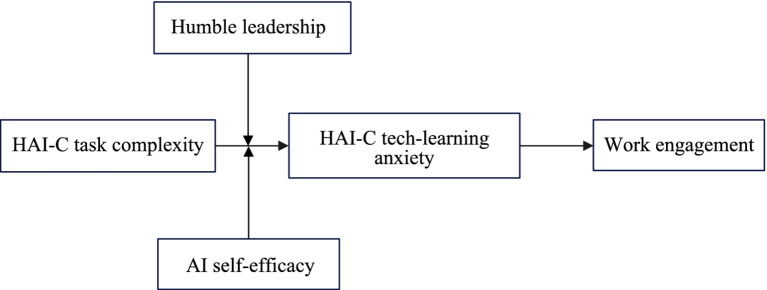
Theoretical model.

AI self-efficacy refers to employees’ subjective perception of their ability to successfully complete tasks related to AI (Hong, 2022; Dong et al., 2025). Employees with high AI self-efficacy, when faced with complex AI collaboration tasks, are driven by their intrinsic beliefs to interpret these high-complexity tasks as manageable challenges. This positive mindset helps alleviate anxiety caused by technological pressure and fosters greater resilience in the face of failure (Parker and Grote, 2022). At the same time, employees with high self-efficacy are more willing to invest personal resources to proactively initiate changes and better adapt to human-AI collaboration (Hong, 2022). For instance, they often actively seek information, persist in learning AI technologies, and adopt proactive coping strategies when encountering complex technical difficulties, rather than resorting to avoidance or emotional depression. These behavioral traits enhance their sense of control in the human-AI collaboration context (Dong et al., 2025; Figure 1) and inhibit the development of HAI-C tech-learning anxiety. Based on this, the following hypothesis is proposed:

*Hypothesis* 5a: AI self-efficacy negatively moderates the relationship between HAI-C task complexity and HAI-C tech-learning anxiety negatively.

*Hypothesis* 5b: AI self-efficacy negatively moderates the mediation of HAI-C tech-learning anxiety between HAI-C task complexity and work engagement.

## 3 Methods

### 3.1 Methodological approach

This study employs a quantitative research method to empirically test the theoretical model. To mitigate common method bias, data were collected through a three-wave questionnaire survey, with one-month intervals between waves, and all variables were self-reported by employees. Core variables were measured using, or adapted from, established and authoritative scales, while demographic and job-related characteristics were controlled. The effective sample size of this study fully meets the regression analysis standards proposed by Green (1991), falling within the ideal range for such analyses.

During the data analysis stage, we adopted a progressive analytical strategy: Confirmatory Factor Analysis (CFA) → Hierarchical Regression Analysis → Hayes’ PROCESS testing. The overall design follows a logic of “progressive deepening, mutual complementarity, and distinct emphasis,” aiming to provide solid and clear empirical support for the research hypotheses. Specifically, the analysis first involved conducting CFA using AMOS to ensure that the structural validity of the measurement model met acceptable standards. Next, hierarchical regression analysis was performed using SPSS. The independent effects of each variable were precisely delineated. This step not only directly tested some of the research hypotheses but also provided preliminary and easily interpretable empirical evidence for subsequent tests of mediation and moderated mediation effects. Finally, the PROCESS macro was utilized to test more complex mechanisms, including mediation and moderated mediation effects. This tool is specifically designed for analyzing conditional process models. Under its default settings, it employs a bootstrap method with 5,000 resamples to calculate 95% confidence intervals for indirect effects. This approach not only allows for more precise and efficient testing of complex mediation mechanisms but also enables the assessment of how mediation effects vary at different levels of the moderator variable. For the complex models involved in this study, such as moderated mediation, PROCESS offers greater statistical power and result robustness. It is also a mainstream method for analyzing mediation and moderation effects in social science fields like management and psychology (Hayes and Rockwood, 2020).

This study leverages the complementary strengths of three methods, thereby methodologically ensuring the reliability and accuracy of the analytical results. Furthermore, cross-validating the same set of hypotheses using multiple methods significantly enhances the robustness of the conclusions. For instance, if hierarchical regression indicates the presence of a mediation effect, and the confidence intervals calculated through the bootstrap sampling method in the PROCESS macro also confirm this finding, the statistical significance of the effect can be substantially affirmed, thereby making the research conclusions more persuasive.

### 3.2 Sample and procedure

This study collected data through the Credamo online platform, a professional survey website in China with a sample pool of over 3 million users. The representativeness of its samples has been validated in recent related studies (Wu and Zhang, 2024; Liu and Li, 2025). Participants in this survey were full-time corporate employees from across China. To ensure voluntary participation, all participants were provided with an informed consent form and assured that their questionnaire responses would be collected anonymously and used solely for academic purposes, thereby alleviating concerns about data leakage. To ensure that participants met the requirement of “collaborating with AI,” we included a screening question at the beginning of the questionnaire: “Do you need to collaborate with AI in your daily work?” Only those who answered “yes” were included as valid samples (Yu L. et al., 2023).

To mitigate common method bias (Podsakoff et al., 2003), this study employed a three-wave research design to test the hypotheses, with each wave spaced 1 month apart. The measurement content at each time point was as follows: In the first wave, questionnaires were distributed to 710 participants, requesting them to evaluate HAI-C task complexity, humble leadership, AI self-efficacy, and provide demographic information. A total of 663 valid questionnaires were collected, with a response rate of 93.38%. In the second wave, questionnaires were distributed to the 663 participants, asking them to evaluate HAI-C tech-learning anxiety. A total of 572 valid questionnaires were collected, with a response rate of 86.275%. In the third wave, questionnaires were distributed to the 572 participants, requiring them to evaluate work engagement. A total of 512 valid questionnaires were collected, with a response rate of 89.51%. After excluding invalid samples with overly short response times (less than 1 min) or clearly patterned responses, a total of 497 valid questionnaires were retained. Participants whose valid questionnaires were adopted at each stage received compensation of 2 Yuan RMB.

The demographic characteristics of the sample are as follows: Regarding gender, males comprised 37.2%, while females accounted for 62.8%. In terms of age, the largest proportion (45.9%) was within the 21–30 age group, followed by the 31–40 age group at 40.8%. For educational background, bachelor’s degree holders constituted the majority at 64.2%, followed by master’s degree holders at 22.3%. Regarding tenure, the largest group (29.2%) had 5–10 years of experience, while both the 0–3 years and over 10 years categories each accounted for 24.5%.

### 3.3 Measurement

Our study directly adopted authoritative scales from prior research. Since the original scales were written in English, we employed Brislin (1970), translation and back-translation method to develop Chinese versions.

HAI-C task complexity was assessed using a four-item scale adapted from Dong et al. (2025). The scale included items such as, “I found the task of working with AI to be a complex task”. Cronbach’s *α* = 0.935 in this study.

HAI-C tech-learning anxiety was assessed using the seven-item scale developed by Wu et al. (2024), which included items such as, “Learning to master all the special skills and methods associated with HAI-C makes me anxious”. Cronbach’s α = 0.897 in this study.

Work engagement was assessed using the nine-item scale developed by Schaufeli et al. (2006), which included items such as, “At my work, I feel like I’m bursting with energy”. Cronbach’s α = 0.932 in this study.

Humble leadership was measured via the nine-item scale developed by Owens and Hekman (2016). One example item was, “This leader actively seeks feedback, even if it is critical”. Cronbach’s α = 0.912 in this study.

AI self-efficacy was measured using a three-item scale adapted from the self-efficacy dimension of Spreitzer (1995) psychological empowerment scale. This scale is widely employed in organizational behavior research to assess individual self-efficacy and has demonstrated strong psychometric properties. To adapt it to the AI work context, we referred to the adaptation approach employed by Dong et al. (2025) for measuring AI self-efficacy. Specifically, we replaced “job” with “job related to AI/working with AI” in the first item of the original scale, changed “activities” to “activities related to AI” in the second item, and substituted “job” with “job related to AI” in the third item. Subsequently, three experts in organizational behavior evaluated the clarity of wording and content validity of the adapted items, ensuring they were easy to understand and free from ambiguity. The scale includes items such as “I am confident about my ability to work with the AI.” Cronbach’s α = 0.729 in this study.

Demographic variables, including gender, age, education level, and tenure, were included as control variables. All scales employed a 7-point Likert response format, ranging from 1 = strongly disagree to 7 = strongly agree.

## 4 Results

### 4.1 Confirmatory factor analysis

Confirmatory factor analysis (CFA) was conducted using AMOS 22.0 software to assess construct validity. As shown in Table 1, the five-factor model demonstrated superior fit indices (χ^2^/df = 2.212, CFI = 0.948, TLI = 0.943, IFI = 0.948, SRMR = 0.049) compared to alternative models, thereby confirming the adequate discriminant validity of the measurement instruments.

**Table 1:** Model fit indices for confirmatory factor analysis.

Model	X^2^	df	X^2^/df	RMSEA	IFI	TLI	CFI
Five-factor model	1002.183	453	2.212	0.049	0.948	0.943	0.948
Four-factor model	2505.088	458	5.470	0.095	0.807	0.790	0.806
Three-factor model	2572.339	461	5.580	0.096	0.801	0.785	0.800
Two-factor model	3322.309	463	7.176	0.112	0.730	0.710	0.729
Single-factor model	5002.049	464	10.780	0.140	0.571	0.540	0.570

*N* = 497. HAI-TC, HAI-C task complexity; HAI-A, HAI-C tech-learning anxiety; WE, Work engagement; HL, Humble leadership; ASE, AI self-efficacy. Five-factor model = HAI-TC, HL, ASE, HAI-A, WE, Four-factor model = HAI-TC, HL, ASE, HAI-A + WE, Three-factor model = HAI-TC, HL, ASE + HAI-A + WE, Two-factor model = HAI-TC, HL + ASE + HAI-A + WE, Single-factor model = HAI-TC + HL + ASE + HAI-A + WE. Source(s): Authors’ work.

### 4.2 Common method bias

Since this study employed a self-report survey methodology, the data may be subject to common method bias. To mitigate the impact of common method bias on the research findings, the following measures were taken:

First, a three-wave time-lagged design was employed during the data collection phase, with core variables measured at three distinct time points. This temporal separation effectively disrupts potential causal thinking habits, transient emotional states, and consistency motivations that respondents might exhibit when answering questions at a single point in time, thereby reducing the interference of common method bias on variable relationship estimation at the research design source.

Second, Harman’s single-factor test was employed to conduct a preliminary screening for common method bias. A principal component analysis was performed on all latent variable items. Among the unrotated extracted factors, the largest eigenvalue factor accounted for only 37.721% of the variance, well below the 50% threshold. This preliminarily suggests that common method bias in this study is limited. However, it should be noted that Harman’s single-factor test is an exploratory tool with limited sensitivity. It can only assess whether a single latent factor dominates the bias based on the data’s variance structure and cannot fully capture common method errors arising from complex factors such as scale design logic or respondents’ social desirability bias. Therefore, these test results serve only as a preliminary reference and cannot independently rule out common method bias.

Third, further analysis combining confirmatory factor analysis revealed that the fit indices of the single-factor model (χ^2^/df = 10.780, CFI = 0.570, TLI = 0.540, IFI = 0.571, SRMR = 0.140) were significantly worse than those of the five-factor model (χ^2^/df = 2.212, CFI = 0.948, TLI = 0.943, IFI = 0.948, SRMR = 0.049). Subsequently, the method of adding an unmeasured common method factor was applied by incorporating a latent variable representing common method bias into the model and comparing the change in model fit. The results showed no significant improvement in model fit after controlling for the common method factor (∆χ^2^ = 269.089, ∆df = 32, ∆χ^2^/∆df = 8.409). In summary, the level of common method bias in this study is within an acceptable range, indicating that the research findings are reliable.

### 4.3 Descriptive statistics and correlation analysis

Descriptive statistics and correlation analyses were conducted on the main variables, and the results are presented in Table 2. Significant correlations were observed between the independent variable (HAI-C task complexity) and the mediating variable (HAI-C tech-learning anxiety), the moderating variables (humble leadership and AI self-efficacy), and the outcome variable (work engagement).

**Table 2:** Descriptive statistics and correlation analysis.

Variable	1	2	3	4	5	6	7	8	9
Gender	1								
Age	−0.056	1							
Education	0.046	−0.228^**^	1						
Tenure	−0.047	0.731^**^	−0.070	1					
HAI-TC	−0.001	−0.113^*^	−0.084	−0.160^**^	1				
HAI-A	0.086	−0.087	−0.193^**^	−0.255^**^	0.320^**^	1			
WE	−0.125^**^	0.166^**^	0.049	0.261^**^	−0.127^**^	−0.465^**^	1		
HL	−0.071	0.055	0.058	0.156^**^	−0.057	−0.351^**^	0.713^**^	1	
ASE	−0.156^**^	0.039	0.153^**^	0.199^**^	−0.183^**^	−0.649^**^	0.604^**^	0.506^**^	1
Mean	1.630	2.650	3.040	2.540	4.423	2.610	5.209	5.537	5.756
SD	0.484	0.824	0.704	1.110	1.524	1.077	1.101	0.903	0.733

*N* = 497, *indicates *p* < 0.05, **indicates *p* < 0.01. HAI-TC, HAI-C task complexity; HAI-A, HAI-C tech-learning anxiety; WE, Work engagement; HL, Humble leadership; ASE, AI self-efficacy. Source(s): Authors’ work.

### 4.4 Hypothesis testing

This study employed hierarchical regression and bootstrapping to examine the mediating effect of HAI-C tech-learning anxiety between HAI-C task complexity and work engagement. The data analysis results are presented in Table 3. Model 1 shows that HAI-C task complexity has a significant positive correlation with HAI-C tech-learning anxiety (*β* = 0.192, *p* < 0.001), supporting Hypothesis 1. Model 6 indicates a significant negative correlation between HAI-C tech-learning anxiety and work engagement (*β* = −0.446, *p* < 0.001), supporting Hypothesis 2. To further examine the mediating role of HAI-C tech-learning anxiety between HAI-C task complexity and work engagement, we applied Hayes’ method using the PROCESS macro in SPSS for conditional process analysis of mediation. Table 4 presents the results of the mediation effect test. As shown, the 95% confidence interval is [−0.104, −0.054], which excludes zero, confirming the existence of the mediating effect. This indicates that HAI-C tech-learning anxiety negatively mediates the relationship between HAI-C task complexity and work engagement, supporting Hypothesis 3.

**Table 3:** Results of hierarchical regression analysis for mediating effects and moderating effects.

Variable	HAI-A				WE	
	Model 1	Model 2	Model 3	Model 4	Model 5	Model 6
Gender	0.192 (0.09) ^*^	0.111(0.085)	−0.011 (0.072)	−0.016 (0.073)	−0.266 (0.098) ^**^	−0.180 (0.09) ^*^
Age	0.199 (0.08) ^*^	0.162 (0.075) ^*^	0.061 (0.064)	0.062 (0.064)	−0.048 (0.087)	0.041 (0.08)
Education	−0.249 (0.065) ^***^	−0.225 (0.06) ^***^	−0.146 (0.052) ^**^	−0.147 (0.052)	0.092 (0.07)	−0.020 (0.065)
Tenure	−0.320 (0.058) ^***^	−0.244 (0.055) ^***^	−0.143 (0.047) ^**^	−0.140 (0.047) ^*^	0.270 (0.063)	0.127 (0.06) ^*^
HAI-TC	0.192 (0.029) ^***^	0.225 (0.029) ^***^	0.180 (0.026) ^***^	0.186 (0.026) ^***^	−0.060 (0.032)	0.026 (0.03)
HL		−0.356 (0.046) ^***^		−0.054 (0.045)		
HAI-TC × HL		−0.133 (0.033) ^***^		−0.028 (0.031)		
ASE			−0.786 (0.052) ^***^	−0.752 (0.060) ^***^		
HAI-TC × ASE			−0.153 (0.039) ^***^	−0.139 (0.042) ^**^		
HAI-A						−0.446 (0.045) ^***^
F	24.064	29.902	69.849	54.555	10.074	26.651
R^2^	0.197	0.300	0.500	0.502	0.093	0.246
Adjust-R^2^	0.189	0.290	0.493	0.491	0.084	0.237

*N* = 497, *indicates *p* < 0.05, **indicates *p* < 0.01, and ***indicates *p* < 0.001. HAI-TC, HAI-C task complexity; HAI-A, HAI-C tech-learning anxiety; WE, Work engagement; HL, Humble leadership; ASE, AI self-efficacy. Source(s): Authors’ work.

**Table 4:** Results of the conditional process analysis for mediating effects.

Path	Effect	BootSE	BootLLCI	BootULCI
HAI-TC → HAI-A → WE	−0.078	0.013	−0.104	−0.054

*N* = 497. HAI-TC, HAI-C task complexity; HAI-A, HAI-C tech-learning anxiety; WE, Work engagement. Source(s): Authors’ work.

To examine the moderating effects of humble leadership and AI self-efficacy, this study first centered the variables HAI-C task complexity, humble leadership, and AI self-efficacy. Interaction terms were then constructed, and hierarchical regression analysis was conducted. The results are also presented in Table 3. As shown in Model 2, the interaction term between HAI-C task complexity and humble leadership has a significant negative effect on HAI-C tech-learning anxiety (*β* = −0.133, *p* < 0.001). This indicates that humble leadership weakens the positive relationship between HAI-C task complexity and HAI-C tech-learning anxiety. Similarly, Model 3 shows that the interaction term between HAI-C task complexity and AI self-efficacy also has a significant negative effect on HAI-C tech-learning anxiety (*β* = −0.153, *p* < 0.001). This suggests that AI self-efficacy similarly weakens this positive relationship between HAI-C task complexity and HAI-C tech-learning anxiety. However, in Model 4 of Table 3, we found that when the interaction term between HAI-C task complexity and humble leadership, as well as the interaction term between HAI-C task complexity and AI self-efficacy, were simultaneously included in the regression model, the effect of the interaction between HAI-C task complexity and AI self-efficacy on HAI-C tech-learning anxiety remained significant (*β* = −0.139, *p* < 0.01). However, the effect of the interaction between HAI-C task complexity and humble leadership on HAI-C tech-learning anxiety became non-significant (*β* = −0.028, *p* > 0.05). This indicates that, after controlling for the variable of AI self-efficacy, humble leadership cannot independently play a moderating role. Therefore, neither Hypothesis 4a nor Hypothesis 4b is supported, while Hypothesis 5a is supported.

**Figure 2:**
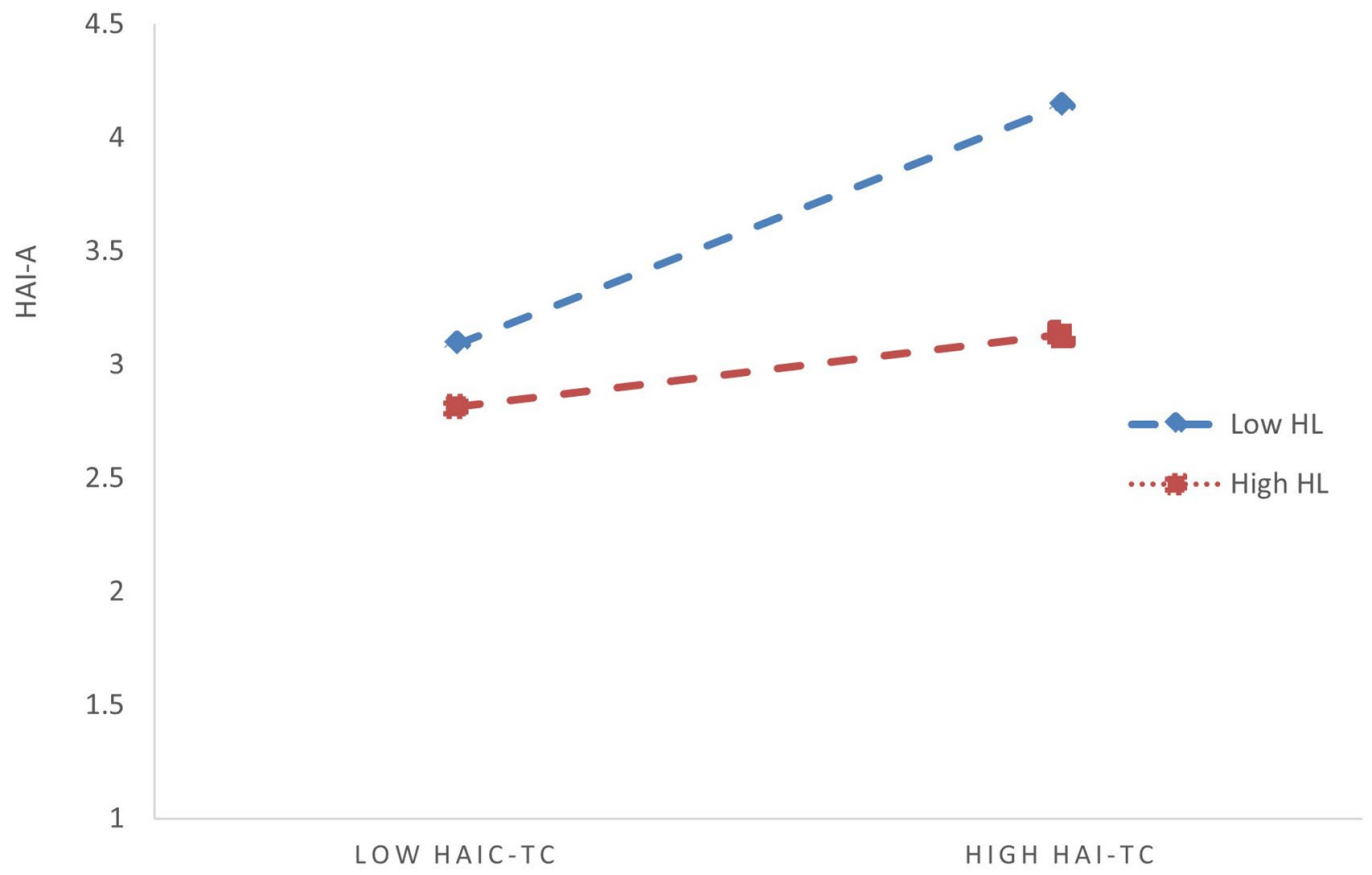
Total moderating effect of HL on the relationship between HAI-TC and HAI-A. HAI-TC, HAI-C task complexity; HAI-A, HAI-C tech-learning anxiety; HL, humble leadership.

**Figure 3:**
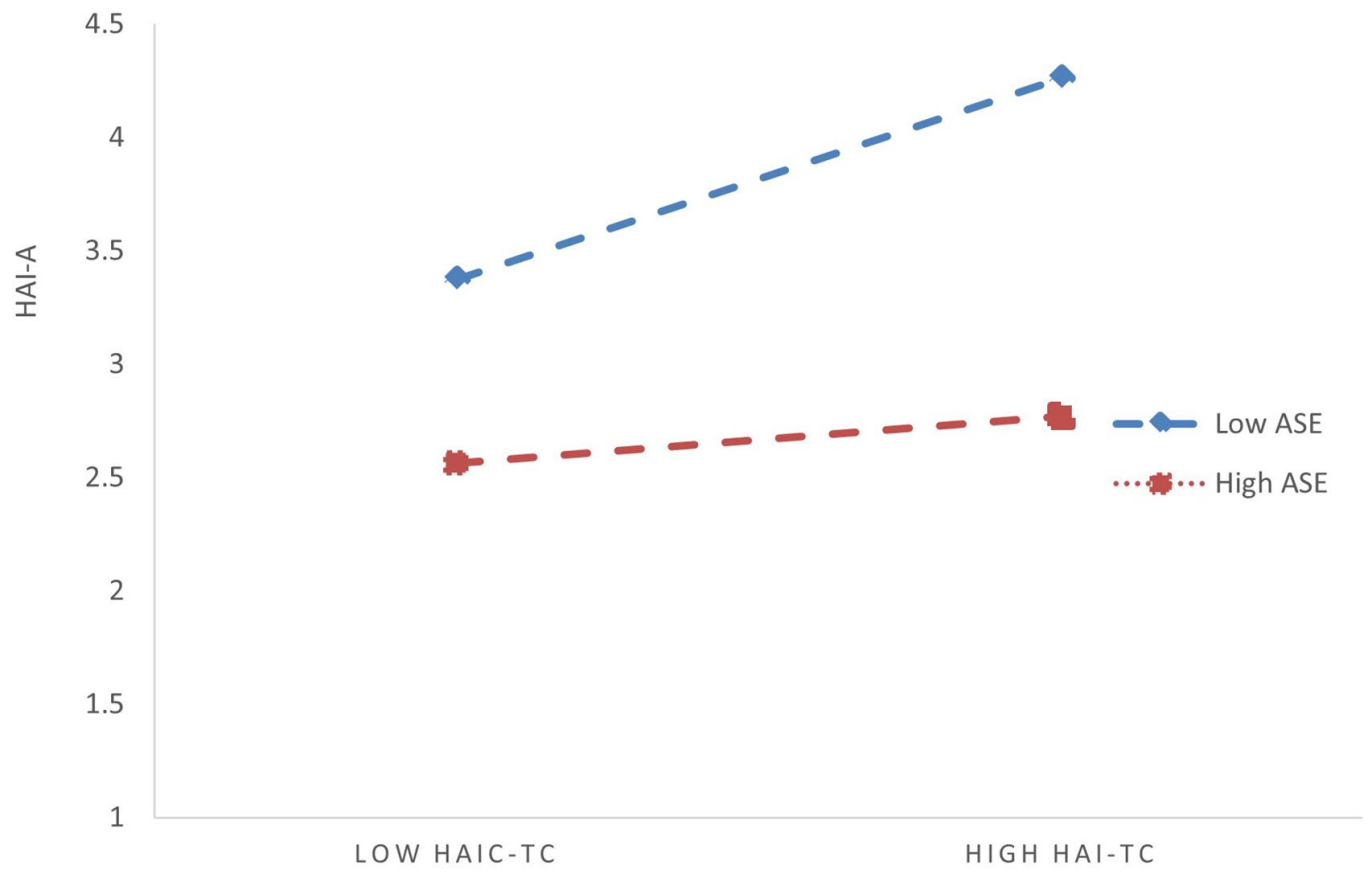
Moderating effect of ASE on the relationship between HAI-TC and HAI-A. HAI-TC, HAI-C task complexity; HAI-A, HAI-C tech-learning anxiety; ASE, AI self-efficacy.

The reason this phenomenon occurs—where both interaction terms are significant when tested separately, but one becomes insignificant when included simultaneously in the regression model—may be due to a correlation between humble leadership and AI self-efficacy. In other words, humble leadership may indirectly moderate the relationship between HAI-C task complexity and HAI-C tech-learning anxiety by influencing AI self-efficacy. To verify this inference, we further examined the predictive effect of humble leadership on AI self-efficacy. The regression results showed a significant positive correlation between humble leadership and AI self-efficacy (*β* = 0.375, SE = 0.030, *p* < 0.001), which supports our inference (Muller et al., 2005). Additionally, from Model 4, we can also observe that after controlling for humble leadership, the independent moderating effect size of AI self-efficacy decreased (from −0.153 to −0.139), and the corresponding *p*-value increased. This demonstrates that the moderating effect of AI self-efficacy is genuine and robust but not entirely independent of humble leadership. Rather, it is positively influenced by humble leadership, meaning that humble leadership provides additional positive reinforcement to the moderating effect of AI self-efficacy. This, in turn, supports the conclusion that humble leadership indirectly moderates the relationship between HAI-C task complexity and HAI-C tech-learning anxiety by enhancing AI self-efficacy. To visually illustrate the initial significant total moderating effect of humble leadership (based on Model 2 in Table 3) and the moderating effect of AI self-efficacy (based on Model 3 in Table 3), we have plotted Figures 2, 3.

To examine the moderated mediation effects of AI self-efficacy between HAI-C task complexity and work engagement, we applied Hayes’ method using the PROCESS macro in SPSS to conduct a conditional process analysis for moderated mediation. Table 5 displays the results of the moderated mediation tests. It can be observed the mediation effect of HAI-C tech-learning anxiety, moderated by AI self-efficacy, has an index of 0.068 with a 95% confidence interval of [0.037, 0.102]. Since this interval does not include zero, it indicates that AI self-efficacy significantly moderates the mediating role of HAI-C tech-learning anxiety between HAI-C task complexity and work engagement. The table also reveals that in the low AI self-efficacy group, the indirect effect of HAI-C task complexity on work engagement is −0.130, with a 95% confidence interval of [−0.177, −0.089]. In the high AI self-efficacy group, the indirect effect of HAI-C task complexity on work engagement decreases to −0.030, with a 95% confidence interval of [−0.050, −0.012]. This demonstrates that the stronger an individual’s AI self-efficacy, the weaker the negative mediating effect of HAI-C tech-learning anxiety, thereby supporting Hypothesis 5b.

**Table 5:** Results of the moderated mediation effect tests.

Mediating variables	Mediator			Index of moderated mediation	
	Moderator	Effect	(CI)	Index	(CI)
HAI-A	Low ASE	−0.130	[−0.177, −0.089]	0.068	[0.037, 0.102]
	High ASE	−0.030	[−0.050, −0.012]		
	Between-Group Differences (High vs. Low)	0.100	[0.055, 0.150]		

*N =* 497. HAI-A, HAI-C tech-learning anxiety; ASE, AI self-efficacy. Source(s): Authors’ work.

## 5 Discussion

**Figure 4:**
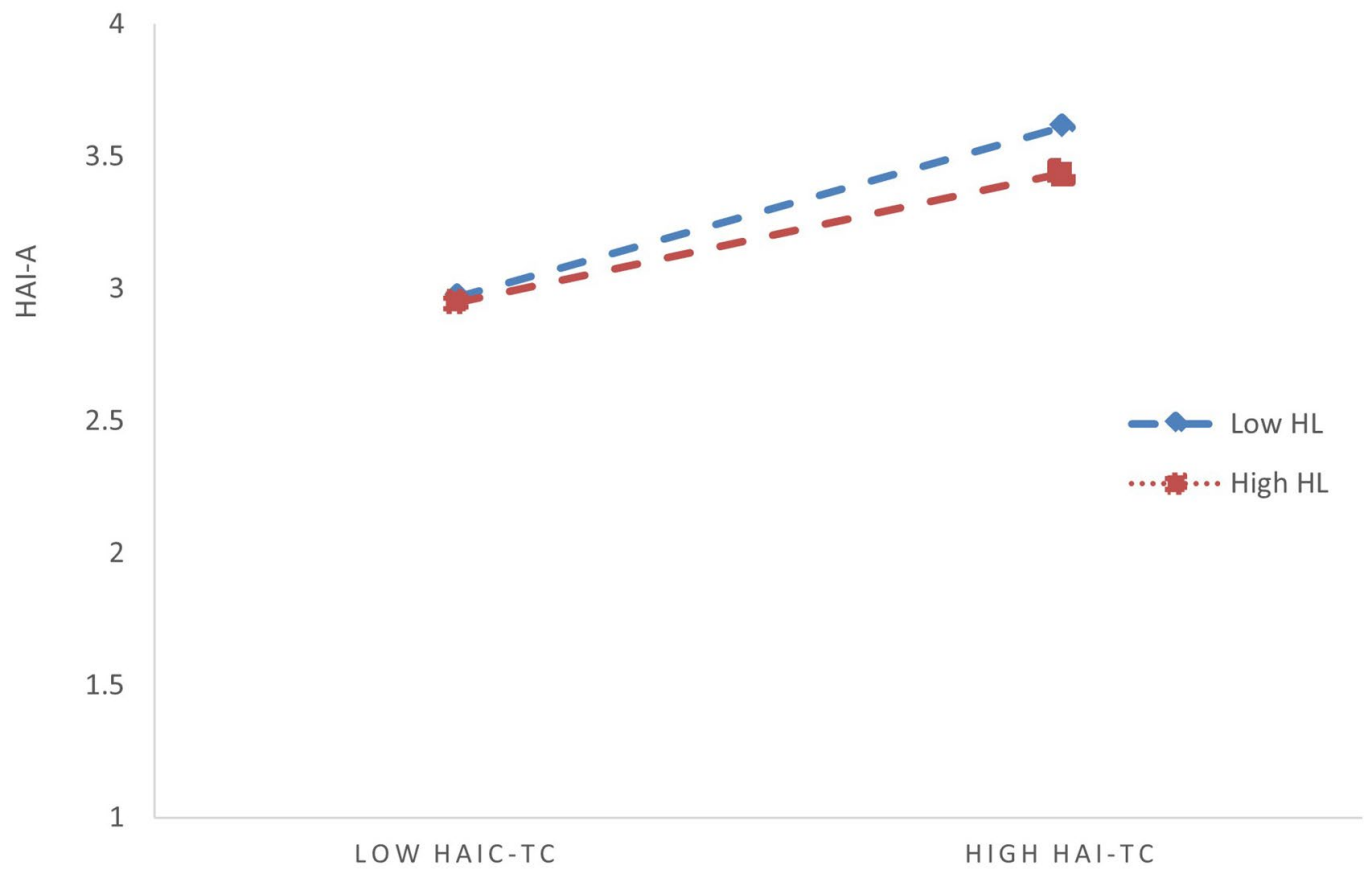
Moderating effect of HL on the relationship between HAI-TC and HAI-A in the full simultaneous model (Model 4 in Table 3). HAI-TC, HAI-C task complexity; HAI-A, HAI-C tech-learning anxiety; HL, Humble leadership.

Based on the JD-R theory, this study confirms the impact of HAI-C task complexity on employees’ work engagement and its intervention mechanisms. The findings indicate that HAI-C task complexity weakens employees’ work engagement by amplifying HAI-C tech-learning anxiety. AI self-efficacy can mitigate the negative indirect effect of HAI-C task complexity on employees’ work engagement (Figure 4).

**Figure 5:**
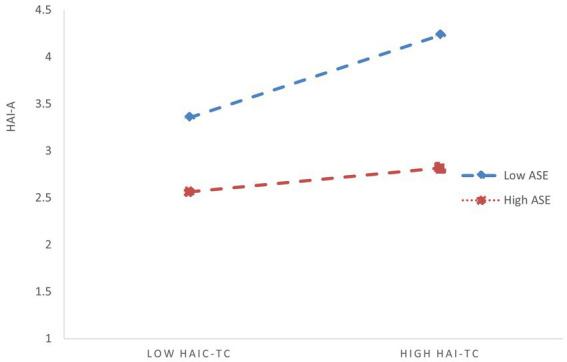
Moderating effect of ASE on the relationship between HAI-TC and HAI-A in the full simultaneous model (Model 4 in Table 3). HAI-TC, HAI-C task complexity; HAI-A, HAI-C tech-learning anxiety; ASE, AI self-efficacy.

Additionally, this study yielded two key findings. First, we found that humble leadership indirectly alleviates this positive effect of HAI-C task complexity on HAI-C tech-learning anxiety by enhancing employees’ AI self-efficacy. In the moderation analysis of humble leadership, when humble leadership was included alone in the regression model, its moderating effect was significant. However, after controlling for AI self-efficacy, its independent moderating effect was no longer statistically detectable. This indicates a certain degree of covariance overlap between humble leadership and AI self-efficacy. Subsequently, we examined the relationship between humble leadership and AI self-efficacy, and the results confirmed a significant positive correlation between the two. This finding provides new insight: humble leadership does not directly moderate the relationship between HAI-C task complexity and HAI-C technology-learning anxiety; instead, it indirectly moderates this relationship by enhancing employees’ AI self-efficacy (Muller et al., 2005). This indicates that job resources do not always directly alleviate the impact of job demands on employees’ strain, sometimes they may produce a mitigating effect by enhancing or transforming into personal resources. On the other hand, when both humble leadership and AI self-efficacy were included in the regression model, the pure independent moderating effect of AI self-efficacy decreased (though still significant). This further verifies that humble leadership provides a positive indirect reinforcement to the moderating effect of AI self-efficacy. In other words, the presence of humble leadership offers a “boosting force” for the moderating effect of AI self-efficacy, amplifying both its effect size and statistical significance. This conclusion also addresses an important proposition of the JD-R theory: in the workplace, job resources and personal resources are not entirely independent; rather, they often interact with each other (Bakker and Demerouti, 2017). In this study, personal resources require the support of job resources to further enhance their buffering effect on work-related stress, while job resources can continuously empower and activate personal resources (Figure 5).

Second, the results indicate that although the negative indirect effect of HAI-C task complexity on employee work engagement is significant, its total effect is not (β = -0.06, *p* > 0.05). This suggests the presence of a potential positive pathway through which HAI-C task complexity influences work engagement. In other words, under certain conditions, HAI-C task complexity may actually enhance employees’ work engagement. This finding aligns with an expanded perspective of the JD-R theory, which posits that an individual’s identification of job demands is shaped by their cognitive appraisal process of these demands (LePine et al., 2005).

With the subsequent development of the JD-R model, researchers have recognized that not all job demands in the workplace are homogeneous. They further distinguished individuals’ perceptions of job demands into two types: challenge job demands and hindrance job demands (LePine et al., 2005; Podsakoff et al., 2007; den Van Broeck et al., 2010). This classification originally stems from the challenge-hindrance stressor framework and has gradually been integrated as a key extension of the JD-R theory (Selye, 1956; Cavanaugh et al., 2000). Challenge job demands refer to work requirements that, although requiring effort, have motivating effects. Hindrance job demands, on the other hand, refer to those that consume energy but hinder goal achievement and fail to provide long-term value (Boswell et al., 2004). Studies have shown that challenge job demands are positively correlated with job satisfaction, motivation, and performance, and negatively correlated with job search behavior and turnover. In contrast, hindrance job demands exhibit the completely opposite pattern of relationships (LePine et al., 2005; Podsakoff et al., 2007).

Early theories posited that an individual’s assessment of job demands is primarily determined by the characteristics of the job itself. However, a growing body of scholars later argued that this assessment does not follow a fixed, uniform pattern. Instead, it is shaped by the combined influence of situational factors and individual traits (Podsakoff et al., 2007). From the perspective of expectancy-value theory, LePine et al. (2004) elaborated on the psychological process through which individuals identify and evaluate job demands. They proposed that the core criteria for an individual’s appraisal of a job demand revolve around three aspects: first, the level of effort required to meet the demand; second, the likelihood of successfully coping with the demand; and third, whether meeting the demand facilitates obtaining desired outcomes (LePine et al., 2004, 2005). Specifically, when individuals perceive that the effort required to meet a job demand is acceptable, the likelihood of fulfilling it is high, and doing so would lead to attaining desirable expected results, they are more inclined to identify that job demand as a challenge demand.

We propose that the non-significant total effect of HAI-C task complexity on employee work engagement can be attributed to AI self-efficacy and humble leadership—acting as individual and contextual factors, respectively—that alter individuals’ cognitive appraisal of HAI-C task complexity. Specifically, as an individual trait, employees with high AI self-efficacy are more inclined to appraise high task complexity in human-AI collaboration as a challenge job demand. This is because such employees with high AI self-efficacy believe they possess, or can quickly acquire, the fundamental cognitive abilities needed to understand AI. When facing complex tasks, they are more confident in formulating effective learning strategies, breaking down complex tasks, and viewing initial difficulties as temporary, surmountable obstacles. Furthermore, they can often foresee more clearly the instrumental benefits of mastering this complex skill. Moreover, for them, the intrinsic satisfaction and sense of competence gained from successfully overcoming a challenge are, in themselves, highly valuable expected outcomes. This also echoes the view of Kilby et al. (2018), that individuals holding positive beliefs about the consequences of experiencing stress are more likely to generate a “challenge appraisal” of job demands. Similarly, as a situational factor, humble leaders excel at inspiring employees’ potential and proactively offering assistance and guidance. This significantly reduces employees’ negative assessment of the effort cost required for tasks and fosters a more optimistic outlook regarding their expectations of success. Simultaneously, their genuine appreciation and recognition of employees’ contributions and growth further enhance the intrinsic value of successful outcomes. Therefore, under the influence of humble leadership, employees’ AI self-efficacy is significantly enhanced and tend to perceive high task complexity in HAI-C as a “manageable challenge that promotes self-actualization.”

Research indicates that when individuals face the challenge job demands, it often triggers positive emotions and prompts proactive or problem-focused coping strategies, such as exerting greater effort (LePine et al., 2005). These demands are typically regarded as possessing attributes of job resources—although they require employees to invest energy, they also hold potential benefits: they can stimulate employees’ curiosity, sense of competence, and conscientiousness, thereby satisfying basic psychological needs, enhancing work vitality, and suppressing the emergence of potential negative emotions (Cavanaugh et al., 2000; McCauley et al., 1994). Consequently, when employees perceive HAI-C task complexity as a challenge job demand, it can instead mitigate HAI-C tech-learning stress and promote increased work engagement. This insight suggests that future explorations into the impact of HAI-C task complexity on employees’ psychology and behavior should move beyond simplistic debates of “good or bad” and instead examine it within specific contexts.

### 5.1 Theoretical contributions

First, this study reveals the psychological mechanisms through which individuals reduce their work engagement when facing HAI-C complex tasks. Existing research on human-AI collaboration has predominantly focused on how this mode enhances work performance, production efficiency, or creativity (Wang M. et al., 2023; Przegalinska et al., 2025; Jia et al., 2024), or has explored its negative impacts on employee psychology and behavior from the perspective of AI substitution (Wu et al., 2024; Kim and Kim, 2024). In contrast, there has been relatively less discussion on the psychological anxiety and behavioral outcomes experienced by employees when confronted with increasingly complex work tasks and technical demands during the human-AI collaboration process. This study addresses and expands upon this research gap. By introducing the JD-R theoretical framework, we demonstrate that individuals involved in human-AI collaboration experience tech-learning pressure due to more complex work tasks, which subsequently leads to lower work engagement. This further enriches the theoretical research related to human-AI collaboration.

Second, existing research on the negative impacts of human-AI collaboration on employees has largely focused on the causes of such behaviors, often overlooking coping strategies and intervention measures at the organizational and individual levels. By applying the JD-R model, this study simultaneously examines two dimensions—job resources and personal resources—and verifying the mitigating effects of AI self-efficacy (active moderation) and humble leadership (indirect moderation) on the pathway through which HAI-C task complexity enhances HAI-C tech-learning anxiety. The conclusions not only provide new insights for organizations and individuals to proactively intervene in and mitigate the negative effects of human-AI collaboration but also further enrich the application of JD-R theory in the field of human-AI collaboration.

Third, the current academic understanding of the conditions under which employees evaluate job demands as either challenges or hindrances remains limited (Mockałło and Widerszal-Bazyl, 2021). This study reveals that when individual AI self-efficacy is sufficiently high, employees are more inclined to identify highly complex tasks in human-AI collaboration as “challenge job demands,” thereby effectively enhancing work engagement (Bakker and Demerouti, 2017). Conversely, when AI self-efficacy is low, the opposite outcome tends to occur. This aligns with the perspective put forward by scholars such as Searle and Auton (2015), Webster et al. (2011), and Bakker and Sanz-Vergel (2013) that “the same job demand may be differentially appraised.” It deepens our understanding of the psychological intervention mechanisms involved in how individuals assess job demands, and further advances the theoretical integration and development of the JD-R theory and the challenge-hindrance stressor framework.

Fourth, existing studies have relatively neglected the role of leadership in intervening employees’ psychological and behavioral outcomes within the context of human-AI collaboration (Bakker and Demerouti, 2017). Humble leadership, as an increasingly valued leadership style in modern organizations (Guo et al., 2025), has an unclear role in the increasingly prevalent human-AI collaboration mode, representing a significant theoretical gap. Based on the JD-R model, this study defines humble leadership as an important job resource and confirms that it mitigates the negative impact of AI collaboration task complexity on employees’ psychology and behavior by enhancing their AI self-efficacy. This enriches research on the relationship between leadership, human-AI collaboration, and employee behavior.

Finally, existing research on the JD-R model remains limited in its interpretation of personal resources. Compared to job resources, the critical role of personal resources in the stress coping process is often overlooked (Schaufeli and Taris, 2014). Moreover, little research has been conducted on the relationship between job resources and personal resources. Therefore, this study introduces “AI self-efficacy” as a core variable, confirming not only its moderating and buffering role in the mechanism through which HAI-C task complexity affects employee work engagement, but also revealing its interrelationship with humble leadership, as well as the internal mechanisms through which both jointly alleviate strain. This finding not only extends the research and application of self-efficacy in the field of human-AI collaboration (Hong, 2022; Wang S. et al., 2023) but also provides an important addition to the discussion in the JD-R theory on how job resources activate personal resources.

### 5.2 Practical implications

First, when employees with low self-efficacy encounter skill challenges in human-AI collaboration, the humble traits of leaders may be particularly crucial. Therefore, for organizations in the early stages of AI adoption (such as enterprises that have recently integrated AI tools into daily operations) or organizations with many employees lacking foundational AI skills, humility should be proactively considered a key dimension in leadership selection and development. For instance, when dealing with AI technology, leaders should be able to openly express curiosity, acknowledge the limits of their knowledge, and share the challenges and insights gained from their own learning processes (Chandler et al., 2023). This approach helps alleviate employees’ lack of self-confidence and anxiety stemming from skill deficiencies. Additionally, when assigning various complex human-machine tasks, leaders should focus more on goals and values rather than adopting an “authoritative stance” by issuing specific directives (Owens and Hekman, 2012). At the same time, leaders should pay attention to employees’ psychological experiences during work, offering both resources and emotional support instead of solely focusing on task outcomes.

Second, in fields that require deep collaboration between employees and AI (such as AI-driven customer service, AI-assisted data analysis, etc.), organizational managers should not implement AI technology in a simplistic or coercive manner. Instead, they should treat the process of employee-AI collaboration as an adaptive process requiring systematic support. For example, a phased training system can be designed to break down complex AI collaboration tasks into progressive skill modules. This allows employees to gradually accumulate successful experiences in collaborating with AI, thereby enhancing their AI self-efficacy, rather than being overwhelmed by an overwhelming demand for skill acquisition all at once.

Finally, regarding skill studying in human-AI collaboration, organizations should focus on fostering a culture and atmosphere that allows for trial and error and encourages seeking help. It is important to clearly communicate the message that “anxiety during the initial learning phase is normal.” Additionally, establishing AI skill workshops led by technical experts or internal mentors can provide employees with clear channels for assistance when encountering difficulties, thereby alleviating their learning anxiety related to AI skills.

### 5.3 Limitations and directions for future research

This study still has the following limitations. First, although a three-phase questionnaire survey method was adopted, since all data were collected through employees’ self-reports, there remains a risk of common method bias (Podsakoff et al., 2012). Future research could employ multi-source survey methods or experimental approaches to investigate related topics, thereby obtaining more rigorous and scientific conclusions. Secondly, while this study confirmed that humble leadership can indirectly moderate the relationship between HAI-C task complexity and HAI-C tech-learning anxiety by enhancing AI self-efficacy, the reason why humble leadership did not produce an independent moderating effect remains unclear. Further investigation is needed to address this issue, aiming to elucidate why Hypothesis 4a and 4b failed to gain support. Finally, regarding the non-significant total effect of HAI-C task complexity on employees’ work engagement, aside from interference from moderating factors, whether other potential positive mediating pathways exist that may form a “suppression effect” alongside the mediating pathway of HAI-C tech-learning anxiety (Wen and Ye, 2014). This issue warrants more comprehensive investigation in future research.

## Data Availability

The raw data supporting the conclusions of this article will be made available by the authors, without undue reservation.
